# What do community paramedics in Germany do regarding the care of older people? A retrospective, descriptive analysis of low-acuity cases

**DOI:** 10.1186/s12873-024-01134-3

**Published:** 2024-11-16

**Authors:** Anna Lena Obst, Insa Seeger, Falk Hoffmann

**Affiliations:** Carl von Ossietzky Universität Oldenburg, School VI – School of Medicine and Health Sciences, Department of Health Services Research, Ammerländer Heerstraße 114-118, Oldenburg, 26129 Germany

**Keywords:** Emergency, Long-term care, Nursing home, Transportation of patients, Health services research, Observational study, Ambulatory care

## Abstract

**Background:**

Non-life-threatening cases treated by emergency services have been increasing in recent years, especially in older people. In a region in Germany with approximately 600,000 inhabitants, the role of a specially trained community paramedic (Gemeindenotfallsanitäter, G-NFS) was introduced in 2019. The G-NFS is dispatched to low-acuity requests, attends the assignment alone and is allowed to treat patients at home.

**Aim:**

The aim of this study was to analyse the assignments attended by the G-NFS relating to the suspected diagnoses, with a focus on older people (≥ 65 years) according to their care setting.

**Methods:**

In this descriptive, retrospective study, we analysed the anonymous assignment report forms 07/2023–12/2023 of cases where patients were aged ≥ 65 years. The suspected diagnoses (free text field) were categorised according to the International Classification of Primary Care 2nd Edition (ICPC-2) scheme. Furthermore, baseline characteristics, urgency of the assignment, provided measures, transport and further treatment were analysed, stratified by care setting.

**Results:**

Of the 1,643 included anonymous assignment report forms, 52.9% (*n* = 869) related to patients aged ≥ 65 years. In this population, the mean age was 80.7 years (SD 8.2), 49.6% were female and most were in long-term care, whether as home care recipients (34.8%) or as nursing home residents (26.9%). The most frequent diagnoses were categorised as urological (24.9%), general and unspecified (13.7%), circulatory (13.6%), digestive (12.8%), musculoskeletal (11.5%) and respiratory (10.3%). In 52.7% of the cases no transport was necessary, while 73.7% of urological cases did not need to be transported.

**Conclusion:**

The G-NFS was dispatched mainly to older people. Most of them were in long-term care and were not transported. The most common suspected diagnoses were categorised as urological, followed by general and unspecified, and circulatory, and differed by care setting. There is a strong need to strengthen outpatient healthcare structures for low-acuity health issues in older and immobile patients.

**Supplementary Information:**

The online version contains supplementary material available at 10.1186/s12873-024-01134-3.

## Introduction

The high number of cases treated by emergency services, including emergency departments (ED) and the emergency medical services (EMS), has posed challenges for healthcare systems worldwide in recent years, especially in industrialised countries [1–3]. As the older age group (≥ 65 years) grows in the future, it is assumed that there will be an ongoing rise in the use of the EMS, as this group has a high need of medical support [4]. The individuals in this group often have non-life-threatening health-related problems, which they take to the EMS [5]. These low-acuity requests could often be treated at home or in outpatient facilities, but if the EMS is dispatched, patients are more likely to be transported to an ED [5, 6]. Accordingly, strategies to relieve overcrowded EDs and overworked EMS must be developed and reevaluated, while focussing on the growing group of older people and their requirements [7, 8]. Community paramedics have been introduced in some countries (e.g. USA, Canada, Finland) [9]. They attend assignments with low-acuity health issues, especially in rural areas, and take care of frequent callers. It has been shown that this part of the EMS might help to save EDs from collapse, reducing costs for the health care system, while still attending to people in need of care [9, 10].

Taking the example of Germany, the number of recorded calls to German rescue control centres (RCC) has risen in recent years and reached almost 17 million calls in 2017 [3]. Various health-related situations prompt patients or caregivers to call the EMS for help, while the number of non-traumatic issues within the field of internal medicine has risen in recent years [11, 12]. It has also been shown that the care of indwelling urinary catheters is a major problem in EMS as it leads to transportation that could have been avoided [13]. In the event of uncertainty or legal concerns, an ambulance must be dispatched to look after the requesting person [13, 14]. Even if the request is in fact not urgent, transportation by the EMS in Germany is a legal obligation to provide the basis for its financing [15]. Older patients make up an important part of this system [16, 17]. As they are often multimorbid, it is difficult to identify if there is an acute problem and more intense efforts are required to treat them [18].

To face the problem of an overcrowded German EMS and avoidable transportation, the resource of the community paramedic (Gemeindenotfallsanitäter, G-NFS) has been developed in a region in Lower Saxony to treat low-acuity requests on an outpatient basis by specially trained paramedics [19]. Previous studies have shown that the introduction of a G-NFS relieves the EMS and avoids transportation [20]. Furthermore, it was found that the G-NFS is dispatched to persons aged ≥ 65 years in 43% of cases and, of these, about six out of ten are care-dependent [21]. However, the clinical picture of the requests treated by the G-NFS could not be identified precisely. To determine the range of requests, suspected diagnoses were recently included in the anonymous assignment report form.

Therefore, the aim of this study was to assess for which suspected diagnoses the G-NFS is dispatched in the case of older people according to their care setting, with a special focus on people who are home care recipients.

## Methods

### Design and Setting

Data in this retrospective, descriptive study are derived from assignment report forms filled out by the G-NFS. We included all patients aged at least 65 years from July 2023 to December 2023. Activities categorised as “first responder” interventions or as incorrect were excluded. First responder cases are categorised as life-threatening by the RCC and the G-NFS is only dispatched if the ambulance is not available in time.

The role of G-NFSs has existed since 2019 and now operates across the geographical regions of Cloppenburg, Vechta and the city of Oldenburg, with about 587,023 inhabitants (3,162 km^2^), situated in the northwest of Lower Saxony, Germany. These territories vary in their structure, encompassing both rural and urban areas. Approximately 25 G-NFSs are active within these areas. The G-NFS ambulance service operates around the clock, with staffing organised into two 12-h shifts. Mobilisation of the G-NFS occurs via the RCC, designated with the European-wide emergency phone number 112. The dispatch system that requests the G-NFS is covered by two RCCs. One is situated in Oldenburg (Großleitstelle Oldenburg) and one in Vechta (Einsatzleitstelle Vechta). The dispatcher at the RCC decides on the acuity according to a standardised system [22]. Thereafter, the dispatcher determines whether to dispatch the EMS, a G-NFS unit for possible outpatient treatment in low-acuity cases or, if it is not urgent, connects to the on-call medical service (phone number 116 117) [14]. The G-NFS attends assignments alone (in contrast to the EMS, which is always sent out with two people), using a vehicle that is equipped with resources tailored to addressing both first responder activities and low-acuity health concerns, including various medications, indwelling urinary catheter sets und urine test strips.

Ethical approval for the study was obtained from the ethics committee of the Carl von Ossietzky University of Oldenburg (2019–030). Since data was collected anonymously, no informed consent was needed.

### Data collection and assessed variables

The one-page assignment report form was designed to assess anonymised information. The form underwent revision in 2023 (version 1.9, appendix) to give more details on suspected diagnoses to provide more accurate information about G-NFS use. The paper-based report forms are subsequently digitised by scanning, with the resultant digital copies forwarded to Carl von Ossietzky University for analysis. Free text fields were evaluated manually.

Information regarding care settings was divided into *no need of long-term care (LTC), home care recipients and nursing home residents*. In Germany, individuals who need LTC can receive benefits from the statutory LTC insurance by submitting an application [14]. Following an extensive and standardised assessment procedure, the Medical Review Board decides on the level of care according to psychological or physical restrictions that are expected to last for at least six months. The care grade required is classified within a system ranging from 1 to 5 (since 2017), with grade 5 indicating the lowest level of independence (physical, psychological) or highest level of dependence on external assistance. The individuals in need of LTC reside either in nursing homes or, in the majority of cases in Germany, in their own homes (84%), receiving care from family members, home care services or from both parties [23].

The free text field on suspected diagnoses was used to assign a corresponding code from the International Classification of Primary Care 2nd Edition (ICPC-2), which was done manually by one of the authors (ALO) based on the manual [24]. The ICPC-2 system categorises diagnoses into 17 distinct groups of symptoms or requests for assistance (A – Z). It was developed for primary care to deal with symptoms that are the reason for the consultation [24]. If multiple diagnoses were suspected, each diagnosis was considered.

Furthermore, fields in the assignment report form regarding baseline characteristics, the measures provided by the G-NFS, use of transportation and further treatment were analysed. Baseline information containing the shift (*day, night*), duration of the operation (*in minutes*) and patient demographics such as sex (*female, male, diverse*), year of birth and previous medical requests for help by the patient (*contacted general practitioner/on-call medical service, no attempt to reach general practitioner/on-call medical service, contact with general practitioner/on-call medical service was not sufficient*) were analysed. Furthermore, urgency was assessed using a part of the *Patientenzuweisungscode* (PZC). This code was originally used by the EMS as feedback to the dispatch system to categorise the urgency and necessity for further treatment and to identify the treatment needed in hospital. Code 1 is for a life-threatening event that needs an intervention immediately, code 2 is for possible inpatient treatment (stay in hospital > 24 h), code 3 is for possible outpatient treatment (stay in hospital < 24 h) and 0 indicates no urgency.

The information about the provided measures was organised as multiple-choice answers and were assessed by ticking a box (*consulting, vital signs, help for self-medication, other medical expertise, urine test stripes, wound care, intravenous access, indwelling urinary catheter care, medication supply, purely nursing measures, other/ increased effort*). Additionally, further information was given by filling out free text fields if necessary (*name of medication supplied, nursing measures, other treatment/ increased effort*).

The information about the requested EMS transportation was assessed by ticking boxes (*no need of transportation, EMS vehicle, add-on call for emergency physician or private transport*). The information about the requested transportation vehicle was combined within one variable (*Transportation by EMS*). We also analysed the advice for further treatment that was given by the G-NFS (*consultation with general practitioner, consultation with on-call medical service, consultation with practising specialist, consultation in emergency department, information to assisting person, other* as free text field).

### Statistical analysis

Descriptive statistics were used. We estimated frequencies and percentages for categorical data and means with standard deviation (SD) for continuous data. All analyses regarding baseline characteristics, ICPC-2 diagnoses, provided measures, transportation and further treatment were stratified by care setting. Additionally, we analysed the requested transportation for the most frequent diagnoses that were given in at least 10% of the cases according to ICPC-2 categories. SPSS (IBM SPSS Statistics Version 29.0) was used for the statistical analysis.

## Results

**Fig. 1:**
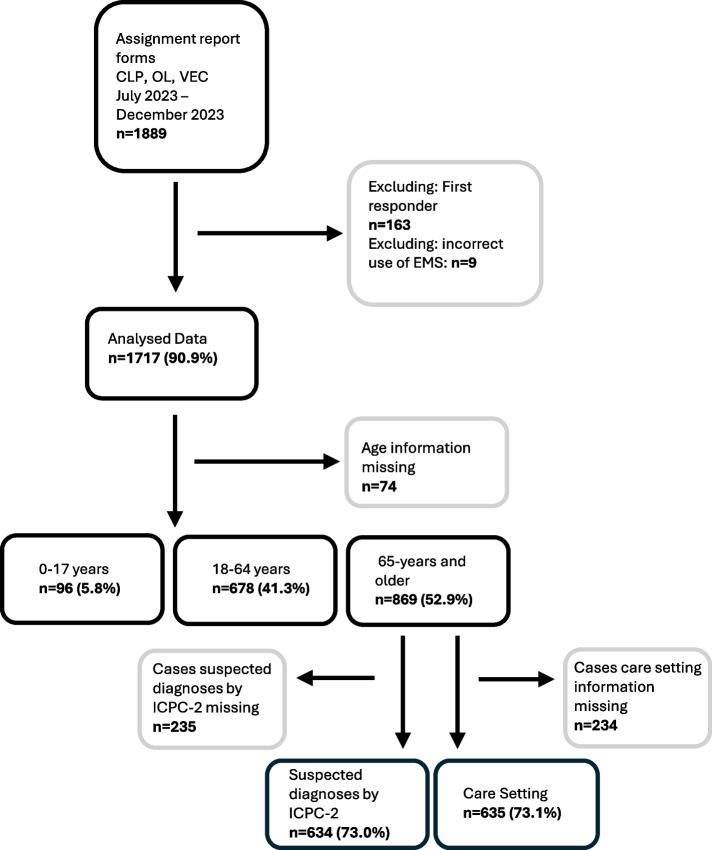
Flowchart of participants aged ≥ 65 years

Of 1,643 reports, 869 cases were included in the analysis as they were aged 65 years and older (52.9%) (Fig. 1). Of those, for 634 cases (73.0%) suspected diagnoses were made that could be categorised according to the ICPC-2 scheme, and a care setting was available in 635 (73.1%) cases.

### Baseline characteristics

In the overall population, the mean age was 80.7 years (SD 8.2), and 49.6% were female (Table 1). Most assignments were carried out during dayshifts (63.1%), with a mean duration of 45.5 min (SD 22.5). More than half of the cases were assessed as not urgent (PZC 0; 54.7%). There was no attempt to contact a general practitioner or the on-call medical service before calling the 112 in 88.1% of cases.

**Table 1:** Baseline Characteristics

	**Overall** **65-years and older**	**No need of LTC**	**Home care recipients**	**Nursing home residents**
**Age in years, Mean (SD)**	***n* = 869**	***n* = 243**	***n* = 221**	***n* = 171**
	80.7 (8.2)	77.5 (7.3)	82.8 (7.7)	83.2 (8.7)
**Sex**^**1**^***n* = , (%)**	***n* = 800**	***n* = 224**	***n* = 202**	***n* = 166**
- Female	397 (49.6)	122 (54.5)	98 (48.5)	71 (42.8)
- Male	403 (50.4)	102 (45.5)	104 (51.5)	95 (57.2)
**Shift *n* = , (%)**	***n* = 849**	***n* = 238**	***n* = 218**	***n* = 167**
- Dayshift	536 (63.1)	150 (63.0)	137 (62.8)	114 (68.3)
- Nightshift	313 (36.9)	88 (37.0)	81 (36.7)	53 (31.7)
**Duration of assignment in minutes, Mean (SD)**	***n* = 865**	***n* = 242**	***n* = 220**	***n* = 170**
	45.5 (22.5)	48.0 (20.1)	46.6 (23.3)	40.3 (18.2)
**Urgency code**^**2**^***n* = , (%)**	***n* = 847**	***n* = 237**	***n* = 213**	***n* = 170**
- PZC 0	463 (54.7)	122 (51.5)	117 (54.9)	107 (62.9)
- PZC 1	47 (5.5)	16 (6.8)	8 (3.8)	8 (4.7)
- PZC 2	203 (24.0)	57 (24.1)	62 (29.1)	24 (14.1)
- PZC 3	134 (15.8)	42 (17.7)	26 (12.2)	31 (18.2)
**Previous use of general practitioner or on-call medical service *n* = , (%)**	***n* = 869**	***n* = 243**	***n* = 221**	***n* = 171**
- Contacted general practitioner or on-call medical service	73 (8.4)	22 (9.1)	22 (10.0)	10 (5.8)
- general practitioner	44 (5.1)	12 (4.9)	13 (5.9)	6 (3.5)
- on-call medical service	29 (3.3)	10 (4.1)	9 (4.1)	4 (2.3)
- No attempt to reach general practitioner or on-call medical service	766 (88.1)	213 (87.7)	193 (87.3)	157 (91.8)
- Contact with general practitioner or on-call medical service was not sufficient	33 (3.8)	11 (4.5)	8 (3.6)	5 (2.9)

^1^Diverse *n* = 0, ^2^Urgency assessed by G-NFS using “Patientenzuweisungscode (PZC)” = Code 1 is for a life-threatening event that needs an intervention immediately, code 2 is for possible inpatient treatment (stay in hospital > 24 h), code 3 is for possible outpatient treatment (stay in hospital < 24 h) and 0 indicates no urgency

Regarding the care setting, most cases were noted as no need of LTC (*n* = 243, 38.3%), then as home care recipients (*n* = 221, 34.8%), then as nursing home residents (*n* = 171, 26.9%). The cases with no need of LTC were younger (77.5 years, SD 7.3) compared with the group of home care recipients (82.8 years, SD 7.7) and nursing home residents (83.2 years, SD 8.7). In addition, the cases with no need of LTC were more often female (54.5%), while there were fewer female cases in the group of home care recipients and nursing home residents (48.5% and 42.8% respectively). If the G-NFS was sent to a nursing home, it was more often a non-urgent request for assistance (PZC = 0, 62.9%) than in the other care settings (54.9%, 51.5%).

### Suspected diagnoses and provided measures

In 634 cases, a diagnosis was suspected by the G-NFS and was categorised using the ICPC-2 (Table 2). In the overall population, most cases were classified as urological (24.9%), followed by general and unspecified (13.7%), circulatory (13.6%), digestive (12.8%), musculoskeletal (11.5%) and respiratory (10.3%). In 869 cases, provided measures were noted, while most frequent measures concerned consulting (81.6%) and vital signs (70.1%). This was followed by medication supply (20.8%), indwellling urinary catheter care (18.1%) and placing an intravenous line (16.5%).

**Table 2:** ICPC-2 categories and provided measures

	**Overall** **65-years and older**	**No need of LTC**	**Home care recipients**	**Nursing home residents**
**Number of suspected diagnoses by ICPC-2 in order to appearance**^a^ ***n* = , (%)**	***n* = 634**	***n* = 164**	***n* = 163**	***n* = 127**
- **U,** Urological	158 (24.9)	14 (8.5)	44 (27.0)	64 (50.4)
- **A,** General and Unspecified	87 (13.7)	20 (12.2)	28 (17.2)	13 (10.2)
- **K,** Circulatory	86 (13.6)	34 (20.7)	22 (13.5)	5 (3.9)
- **D,** Digestive	81 (12.8)	30 (18.3)	19 (11.7)	13 (10.2)
- **L,** Musculoskeletal	73 (11.5)	27 (16.5)	15 (9.2)	6 (4.7)
- **R,** Respiratory	65 (10.3)	23 (14.0)	10 (6.1)	14 (11.0)
- **P,** Psychological	30 (4.7)	7 (4.3)	11 (6.7)	1 (0.8)
- **T,** Endocrine, metabolic and nutritional	27 (4.3)	9 (5.5)	8 (4.9)	5 (3.9)
- **N,** Neurological	25 (3.9)	12 (7.3)	5 (3.1)	1 (0.8)
- **S,** Skin	25 (3.9)	5 (3.0)	5 (3.1)	8 (6.3)
- **Z,** Social problems	17 (2.7)	2 (1.1)	9 (5.5)	2 (1.6)
- **B,** Blood, blood forming organs and Immune Mechanism	1 (0.2)	1 (0.6)	0	0
- **X,** Female genital	4 (0.6)	0	2 (1.2)	1 (0.8)
- **H,** Ear	3 (0.5)	1 (0.5)	0	1 (0.8)
- **Y,** Male genital	3 (0.5)	0	0	2 (1.6)
- **F,** Eye	1 (0,2)	0	0	1 (0.8)
- **W,** Pregnancy, childbearing, family planning (Women)	0	0	0	0
**Measures in order to appearance**^**a**^ ***n* = , (%)**	***n* = 869**	***n* = 243**	***n* = 221**	***n* = 171**
- Consulting	709 (81.6)	218 (89.7)	193 (87.3)	119 (69.6)
- Vital signs	609 (70.1)	192 (79.0)	162 (73.3)	95 (55.6)
- Medication supply (GNFS)	181 (20.8)	63 (25.9)	39 (17.6)	29 (17.0)
- Indwelling urinary catheter care	157 (18.1)	6 (2.5)	43 (19.5)	67 (39.2)
- Intravenous line	143 (16.5)	56 (23.0)	32 (14.5)	22 (12.9)
- Help for self-medication	101 (11.6)	36 (14.8)	33 (14.9)	10 (5.8)
- Other/ Special features/ increased effort	73 (8.4)	20 (8.2)	32 (14.5)	9 (5.3)
- Urine test stripes	32 (3.7)	4 (1.6)	13 (5.9)	7 (4.1)
- Other medical expertise	23 (2.6)	4 (1.6)	8 (3.6)	3 (1.8)
- Purely nursing measures	19 (2.2)	2 (0.8)	11 (5.0)	3 (1.8)
- Wound care	17 (2.0)	4 (1.6)	6 (2.7)	3 (1.8)

^a^Multiple answers possible

The most frequent ICPC-2 diagnoses varied depending on the care setting. While urological issues were most often present in people who were in need of LTC, in nursing home residents (50.4%) and home care recipients (27.0%), this was a less relevant request in the group with no need of LTC (8.5%). In the latter group, most requests were categorised as circulatory, followed by digestive and musculoskeletal. Regarding the home care recipients, the most common requests after urological issues were for general and unspecified, and circulatory issues. In spite of this, nursing home residents frequently showed respiratory, general and unspecified, and digestive requests.

Consultation (81.6%) and vital signs (70.1%) were the leading tasks across the population, although less so for nursing home residents (69.6% and 55.6% respectively). These were followed by medication supply, which was provided in 20.8% of the overall population and in the group with no need of LTC (25.9%). In the group of home care recipients this was 17.6% and in nursing home residents 17.0%. Furthermore, indwelling catheter care was a frequently provided measure in the overall population (18.1%). In nursing home residents it was provided in 39.2% of cases, in home care recipients in 19.5% of cases, while it was provided in 2.5% of cases in the group with no need of LTC. In the overall population, an intravenous line was placed in 16.5% of cases. In the group with no need of LTC, it was placed in 23.0% of cases, while it was placed in 14.5% of cases in home care recipients and in 12.9% of cases in nursing home residents.

### Transportation and further treatment

Regarding the overall population, transportation was not necessary in most cases (52.7%) (Table 3). The next most common category was transportation by EMS (39.4%). The G-NFS advised a consultation in the ED in 42.0% of cases and a consultation with the general practitioner in 38.2% of cases.

**Table 3:** Transportation and advice for further treatment

	**Overall** **65-years and older**	**No need of LTC**	**Home care recipients**	**Nursing home residents**
**Transport** ***n* = , (%)**	***n* = 869**	***n* = 243**	***n* = 221**	***n* = 171**
- No transportation	458 (52.7)	118 (48.6)	121 (54.8)	108 (63.2)
- Transportation by EMS^1^	342 (39.4)	97 (39.9)	87 (39.4)	60 (35.1)
- Add-on call for emergency physician	20 (2.3)	6 (2.5)	5 (2.3)	4 (2.3)
- Private transportation	43 (4.9)	19 (7.8)	6 (2.7)	0
**Advice for further treatment**^a^ ***n* = , (%)**	***n* = 896**	***n* = 243**	***n* = 221**	***n* = 171**
- Consultation with the general practitioner	332 (38.2)	109 (44.9)	82 (37.1)	54 (31.0)
- Consultation with the on-call medical service	41 (4.7)	13 (5.3)	7 (3.2)	6 (3.5)
- Consultation with practicing specialist	74 (8.5)	10 (4.1)	26 (11.8)	26 (15.2)
- Consultation in the ED	365 (42.0)	113 (46.5)	89 (40.3)	57 (33.3)
- Information given to assisting person (i.e. nurse or relatives)	75 (8.6)	5 (2.1)	30 (13.6)	21 (12.3)
- Other (i.e. Psychiatry, free text)	39 (4.5)	10 (4.1)	11 (5.0)	10 (5.8)

^a^Multiple answers possible, ^1^EMS = combined variable of differently qualified two-staffed EMS vehicles (Rettungswagen = RTW *n* = 108, (31.6%), Notfallkrankenwagen = NKTW, *n* = 118, (34.5%), Krankentransportwagen = KTW, *n* = 116, (33.9%) regarding overall population (all persons aged 65-years and older)

The most common option was no need of transportation in all care settings, with the proportion increasing from no need of LTC (48.6%), to home care recipients (54.8%) to nursing home residents (63.2%). Accordingly, the advice to get a consultation at the ED increased from nursing home residents (33.3%), to home care recipients (40.3%), to no need of LTC (46.5%). The same tendency was shown with the advice to get a consultation with the general practitioner.

**Fig. 2:**
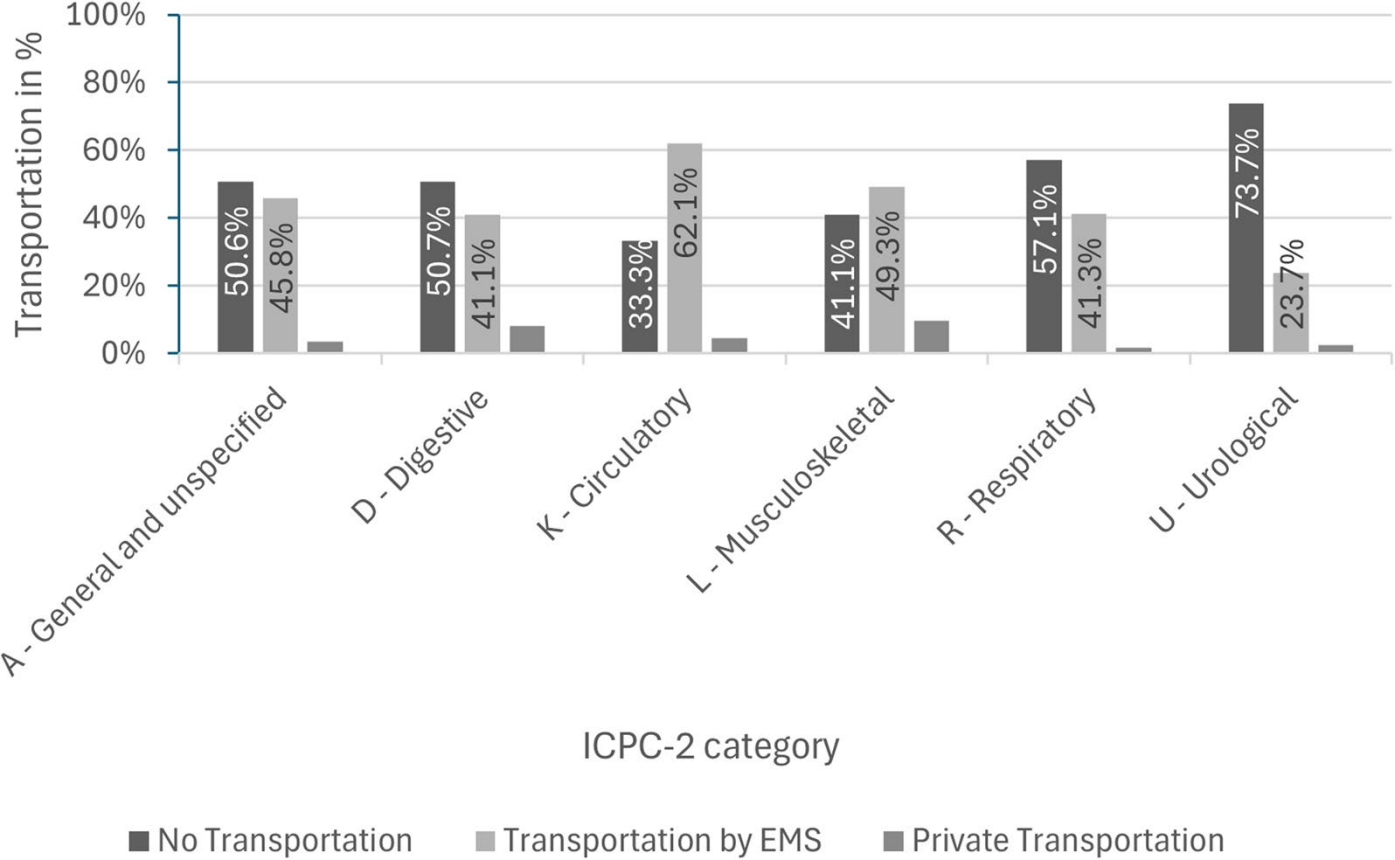
Transportation in terms of ICPC-2 categories (> 10%), *n* = 535

In the analysis of the type of transportation based on the suspected diagnoses according to the ICPC-2 that were classified in at least 10% of the cases, 535 cases were evaluated (Fig. 2). This included the categories urological (U), respiratory (R), general and unspecified (A), digestive (D), circulatory (K), and musculoskeletal (L). Of the cases categorised as U, 73.7% did not need to be transported. Furthermore, in the categories R, A and D, most cases did not need to be transported. Only the categories K and L lead to more EMS transportation than no transport (62.1% and 49.3% respectively).

## Discussion

In our study, more than half of the assignment report forms (52.9%) related to the population of older people (65 years and older). In this population, 6 out of 10 were care-dependent, whether as home care recipients or as nursing home residents. These cases showed less urgent requests and most of them were not transported. The most frequent ICPC-2 diagnoses differed between care settings. Overall, 24.9% of the cases were categorised as urological (8.5% in those with no need of LTC, 27.0% in home care recipients and 50.4% in nursing home residents). A large proportion of the urological cases (73.7%) did not need to be transported. People who were not in need of LTC showed more urgent requests, compared with people who were in need of LTC. Additionally, they had more cases categorised as circulatory (20.7%), digestive (18.3%) and musculoskeletal (16.5%).

### Emergency medical care in older people

The G-NFS was dispatched frequently to older people (52.9%). This proportion is even higher than in comparable study populations of older patients in EDs [5, 12, 25]. Moreover, our findings are consistent with existing literature recounting a growing proportion of older people using emergency services [17, 25]. The study population had a mean age of 80.7 years. Roessler et al. also showed a peak in use of EMS in the 80 to 90 year age group, who were mainly nursing home residents [5]. The G-NFS was dispatched to nursing home residents in 26.9% of cases and to home care recipients in 34.8% of cases. The majority of our study population was therefore in need of LTC. According to the Federal Statistical Office of Germany (Destatis) the number of people in need of LTC will rise in the coming years due to demographic changes [26]. This means that although at the end of 2021 there were about 5 million people in need of LTC in Germany [23], it is estimated that this number will increase to 6.8 million people in need of LTC by 2055. [26]. The expected and predicted increase in older and immobile patients is challenging the healthcare system. These patients would benefit from care being supplied with minimal effort, preferably at home. However, in recent years, the trend shows that home visits by general practitioners are decreasing, especially in rural areas [27, 28]. Our study showed that 88.1% of cases did not contact their general practitioner before calling 112, but the G-NFS nevertheless advised a consultation with the general practitioner in 38.2% of cases. Overall, the pathways of emergency treatment and outpatient structures for non-life-threatening requests in Germany are difficult to follow for patients and have several hurdles for older, immobile patients (i.e. they are not barrier-free) [14].

### ICPC-2 categories—Urological needs

People in need of LTC frequently presented requests in the urological category, with nursing home residents representing more than half of cases. Conversely, the urological category was not a major request in the cases who were not in need of LTC (8.5%). Our study also showed that the proportion of male cases was higher in nursing home residents (57.2%), even though more women live in nursing homes [29]. Additionally, the provided measures showed that indwelling urinary catheter care was provided in 39.2% of nursing home residents and most of them were male. Our data also showed that 73.7% of the urological cases did not have to be transported. In line with this, Heinold et al. found that catheter problems form a common reason for mainly male nursing home residents to be transferred to an ED for an ambulant treatment [30]. Furthermore, they found that the treatment was mostly about easy interventions (“changing an existing catheter or flushing the pre-existing one”). The residents were transferred back to their nursing home (by EMS) after a treatment that could have been done as an outpatient as well. This clearly shows that these catheter-related transports to hospital were not necessary, which was underlined by the qualitative study of ambulance staff by Pulst et al. [13]. Interviews with the G-NFS by Sommer et al. supported these findings [31]. There the trained G-NFS categorised the skill of taking care of catheters as a helpful addition to their initial training. They reported that this new skill forms a possible solution to relieve the EMS. Concurrently, this shows that there is a challenge for the healthcare system regarding older and often immobile patients with indwelling catheters, especially in nursing homes. It underlines that the G-NFS is an important addition to the existing care supply structures. As this project is unique in Germany, and therefore limited to the described region, further studies in other areas would be helpful to gather a broader picture on indwelling catheter care and catheter-related transportation.

Moreover, a notable number of cases were categorised as circulatory (13.6%). In the group who were not in need of LTC, this category had the highest proportion with 20.7%. As studies already showed, cases relating to internal medicine are an increasing reason for calling the EMS [3]. We also showed that circulatory requests were more likely to be transported (62.1%). The urgency of this category was also seen in findings in an out-of-hour-office in Norway [32]. In their study Raknes and Hunskaar found that the category circulatory, chest pain, was a common indication for urgent triage (red). Furthermore, they found that general and unspecified issues, as well as musculoskeletal issues, were frequent reasons for contact in this primary care setting. According to our findings, it should be discussed whether the RCC should dispatch a transport vehicle instead of a G-NFS in these cases.

### Strengths and Limitations

A strength of this study was the high number of assignment report forms that were collected over the period of half a year. As the number of respective EMS reports is quite comparable, we assume that nearly every assignment had a report. These reports recount important information about the care setting, which is not available in respective EMS reports. In our study, information about the care setting was given in 73.1% of cases and about suspected diagnoses in 73.0% of the assignment report forms. Some missing data in the category of suspected diagnoses might be explained by requests that were not healthcare-related and therefore not written down by the G-NFS. However, in most cases a suspected diagnosis was noted and could be categorised according to the ICPC-2. The ICPC-2 categories were developed for primary care consultations, cover physical, mental and sociological conditions [33] and fitted best to our study population. In addition, some missing data may be due to individual G-NFSs not filling the form to the appropriate level of completeness. The G-NFS fills out the paper-based assignment report form during their daily work schedule and we cannot gather information about the precision of the given information. It might be filled in after the assignment, and unknown information may be completed according to the G-NFS’ perception by memory. As the cases are anonymous, we cannot tell if the patient called the RCC once more or went to the ED even if it was not advised. Moreover, the advised treatment is not explained further. The assignment report form does not provide information as to whether a visit to the ED was recommended by the G-NFS due to the use of a special diagnostic tool (i.e. X-Ray) or if an inpatient treatment was expected.

## Conclusion

This study showed that 6 out of 10 cases aged ≥ 65 years attended by the G-NFS were care-dependent and more than half of them were treated as an outpatient without being transported. The suspected diagnoses according to ICPC-2 were mainly categorised as urological, followed by general and unspecified, and circulatory, but varied by care setting. Urological problems did not need to be transported in 7 out of 10 cases. There seems to be a lack of outpatient medical structures for older, immobile patients in Germany. As this population will increase in the coming years, there is a strong need to develop and strengthen outpatient healthcare structures to deal with such low-acuity health issues. Future research should focus on outpatient care for older, immobile patients with a special focus on the care of indwelling urinary catheters.

## Supplementary Information

Supplementary Material 1.

## Data Availability

The datasets used and/or analysed during the current study are available from the corresponding author on reasonable request.

## Abbreviations

G-NFS: Gemeindenotfallsanitäter (community paramedic)
ED: Emergency department
EMS: Emergency medical service
RCC: Rescue control centre
LTC: Long-term care
ICPC-2: International Classification of Primary Care 2nd Edition
