# Supplementary material for: What do community paramedics in Germany do regarding the care of older people? A retrospective, descriptive analysis of low-acuity cases
Source: BMC Emerg Med. 2024 Nov 16;24:215. doi: 10.1186/s12873-024-01134-3 (PMC11569594; doi:10.1186/s12873-024-01134-3)
Supplement: Supplementary file 1 — Supplementary Material 1. [file 12873_2024_1134_MOESM1_ESM.pdf]

**Supplement 1: Basis for the assignment report form filled out by G-NFS**

| | |
|---|---|
| **Supplement methods** | |
| **Demographic data** | |
| Year of birth, sex | |
| **Other** | |
| City (Landkreis), Date, dayshift (7am-19pm), nightshift (19pm-7am), first responder operation, support for ambulance | Single choice |
| **Clinical picture** | |
| Emergency call notification RCC*, suspected diagnoses*, categorisation of diseases: CNS, cardiovascular system, respiratory, abdominal, psychiatry, metabolism, paediatrics, gynaecology, urology, surgical, other | *Free text field, multiple choice |
| **Care setting (only for pat. ≥65 years)** | |
| No need of LTC, nursing home resident, home care recipient by: family member, home care services, care grade* | Single choice, *free text field |
| **PZC (Patientenzuweisungscode) = urgency** | |
| PZC 0: no urgency, no transportation needed<br>PZC 1: life-threatening, immediate intervention is needed<br>PZC 2: suspected inpatient treatment (stay in hospital >24h)<br>PZC 3: suspected outpatient treatment | Single choice |
| **Provided measures** | |
| consulting, vital signs, help for self-medication, other medical expertise, urine test stripes, wound care, intravenous line, indwelling urinary catheter care apply/ remove/ rinse, medication supply (G-NFS)*, purely nursing measures*, other/ increased effort* | Multiple choice, *free text field |
| **Utilisation telemedicine** | |
| Was used: Klinikum Oldenburg, on-call medical service/ 116117, other, telemedicine was not used | Multiple choice |
| **Requested EMS transportation** | |
| No need of transportation, ambulance RTW[1] , add on call for emergency physician, ambulance NKTW[2], accompaniment of emergency transport, NKTW/ MZF[4] not available, RCC dispatched: RTW/ KTW, KTW[3] not available, RCC dispatched: RTW, NKTW/MZF, private transport, Taxi/ rental car, wheelchair transport, request GNFS by RTW/N-KTW/KTW, Taxi could have replaced ambulance (KTW/RTW/NKTW) | Single choice |
| **Advice for further treatment** | |
| consultation: general practitioner, on-call medical service, practicing specialist, emergency department, psychiatry/psych. on-call service, information towards nursing service (ambulant/ nursing home), towards family members, crisis intervention, consultation psychiatry/psych. on-call service | Multiple choice |
| **Preceding medical request used by patient** | |
| contacted general practitioner/ on-call medical service, no attempt to reach general practitioner/ on-call medical service, contact with general practitioner/ on-call medical service was not sufficient: overcrowded, advice to call the 112, could not be reached | Multiple choice |
| **Assessment by GNFS about categorisation done by RCC** | |
| Assessed correctly, urgency was dispatched too high or too low, duration of operation (minutes), G-NFS training was adequate for this operation: yes, no: further training in: | Single choice |

**Abbreviation:**

CNS= central nervous system

LTC= long-term care

RCC= rescue control centre

**Ambulance in Germany**:

[1]Two-staffed, highest qualification = Rettungswagen (RTW)

[2]Two-staffed, second highest qualification = Notfallkrankentransportwagen (NKTW)

[3]Two-staffed, lowest qualification = Krankentransportwagen (KTW)

[4]Two-staffed, can be adapted to urgency of emergency call (similar to NKTW) = Mehrzweckfahrzeug (MFZ)
